# Autopsies at Central Park Menagerie

**Published:** 1885-10

**Authors:** 


### CASE DEPARTMENT.

### I.—AUTOPSIES AT CENTRAL PARK MENAGERIE.

Tiger (*Felis tigris.*) *Hab. India.*—Æt. 10. The tiger had been in labor fifty-eight hours. Two young ones had been delivered by manual extraction with difficulty, the bony pelvis being too much contracted for a normal labor. Both of these young ones were born dead. The membranes had not come away, nor the placentæ. Four hours after the extraction of the second young one the animal showed signs of exhaustion, and died at 10 P.M.

Autopsy ten hours after death.

The body was well nourished, not fat; rigor mortis moderate. The abdomen was much distended. There was some external hæmorrhage from the vulva.

On opening the abdomen a large amount of gas escaped, allowing the wall to collapse. As the opening was enlarged a considerable amount of blood, which was lying free in the abdominal cavity, escaped. The amount could not be measured, but was at least four quarts.

The liver, spleen, stomach and intestines appeared to be normal.

The kidneys were not examined. The uterus was about twenty inches in length, its wall was thin and relaxed, and the organ was evidently distended by a fœtus. At its upper portion, on the right side of the fundus, just ventral of the entrance of the fallopian tube, a small rupture of the wall was found, from which blood could be pressed out, and through which one paw of the fœtus protruded. It was evident that the source of the hemorrhage into the abdomen had been the ruptured arteries. The uterus contained one fœtus in its membranes, partly decomposed, the placentæ of the two which had been born, and considerable amount of fœtal blood. Its inner wall was black, soft and fetid. There was no peritonitis.

The bony pelvis was not dissected out, but the inability to introduce more than two fingers of the hand through it indicated the existence of a contraction.

Lungs entirely collapsed, crepitated on pressure, and on cutting contained very little blood; no œdema. They were deeply pigmented. There was no consolidation, and no tubercles. No adhesions between the pleural surfaces.

Heart firm—contracted in systole; ventricles empty. No pericarditis. Brain normal.

M. Allen Starr, M.D., Ph. D.

*Cause of Death in Parrots.*—On two occasions we have had an opportunity of examining large Ara parrots found dead in the aviary, without being able to discover the cause of death. In the first of these cases, the bird had suddenly fallen from its perch. The viscera were healthy, the gizzard pale and empty, and the only morbid condition found was a congestive suffusion of the fascia and other tissues of the neck, and a normal softness of the cerebral tissue, with congestion of the cervical and cerebral medulla. All the appearances may be due to mortuary causes of very different origin. It is not improbable that some circulatory trouble may be at the bottom however. The parrot has—so-to-speak—an overgrown head, and this contains an enormous brain, requiring a large proportion of the blood in the body for its nourishment. The neck, however, is thin. the carotids of small calibre, and consequently the heart is compelled to work under some disadvantage, in order to overcome the combined factors of length and narrowness of the blood-conduits. The large cerebral vein which runs in the dorsal sulcus of each hemisphere was, in both of the mentioned cases, found filled with a firm black coagulum.

In connection with this fact, we desire to call attention to the generally unsatisfactory state of bird pathology, which is due, no doubt, to special difficulties in the way of impalpable lesions. Thus while the reporter was home, alone one afternoon, a pigeon flew in through the window, and alighted on the top of a door which was fully open, and remained here three hours, and was undisturbed, in order that it might accommodate itself to the location and become a pet for the little boy. Food was scattered for it, which it did not touch; but aside from the fact—which was singular enough—that it had flown into a strange house, without seeming to be aware of it, nothing unusual was noted, Suddenly the reporter was startled by a noise, and the animal was found on the ground in a fluttering fit; from this it recovered, for a few minutes, but was noticed to twist its head around to the right, and to flutter in a circle. It then had a violent epileptiform convulsion, and this continuing with unremitting severity, its neck was wrung to end its misery. Nothing abnormal was found, except that when the brain was removed a circular area on the right hemisphere appeared to sink in a litle, and showed a slightly softer consistency than the remainder, so that agitating it under a gentle stream of water, this part dissolved and came away. ***

———

American Crow (*Corvus Americanus*)—received, September 14th. All the viscera structurally healthy. Some coagulated blood occluded the nasal orifices, and the head and neck feathers were found matted together by smaller coagula and serum. A punctured wound was found, two lines to the right of the median line, on the most prominent part of the cranial convexity. This wound was cleanly cut and triangular; a corresponding aperture extended through the bone. On cutting through the cranium, to expose the brain, the cancellated tissue, which normally is of a pure white color, and empty, was found filled with blood. A slight coagulum extended over the cerebellum and the right cerebral Dura. The latter was lacerated at the posterior declivity, in a longitudinal rent, and the corresponding portion of the cerebrum was clearly cut in the same downward direction. The wounded part of the brain exhibited eversion of the edges (inflammatory swelling) and softening. The entire convexity of the right hemisphere exhibited beautifully marked lesions. There were eight oval black spots in a group, from the size of a pin's stem to a pin's head, near the sagittal vascular enleus; a group of three similar and smaller spots was found further forward in the same line. Interspersed with these dark spots, which were blood coagula, there were smaller red dots, and towards the lateral part of the hemisphere these merged into an intensely injected area. The rest of the brain was unusually pale, that is anæmic, even the great cerebral vein being empty.

Anatomico-pathological diagnosis: *Vulnus ceribri*, complicated by ceribritis, filling of the cranial air spaces with blood—consequent asphyxia.

The interesting feature of the case is the demonstration of the effect of violent blows on the bird's brain, namely, the production of numerous minute capillary hemorrhages, *both from the arterial and venous capillaries.* These hemorrhages were none of them on the surface, but imbedded immediately beneath it, in the cerebral tissue proper. The violence of the concussion to which the animal was subjected was shown by the fact that the cranio-mandibular suture was loosened. ⁂

